# Supplementary material for: hReg-CNCC reconstructs a regulatory network in human cranial neural crest cells and annotates variants in a developmental context
Source: Commun Biol. 2021 Apr 6;4:442. doi: 10.1038/s42003-021-01970-0 (PMC8024315; doi:10.1038/s42003-021-01970-0)
Supplement: Supplementary file 2 — Supplementary Information [file 42003_2021_1970_MOESM2_ESM.pdf]

Supplementary Information

hReg-CNCC reconstructs a regulatory network in human cranial neural crest cells and annotates variants in a developmental context

Zhanying Feng, Zhana Duren, Ziyi Xiong, Sijia Wang, Fan Liu*, Wing Hung Wong*, Yong Wang*

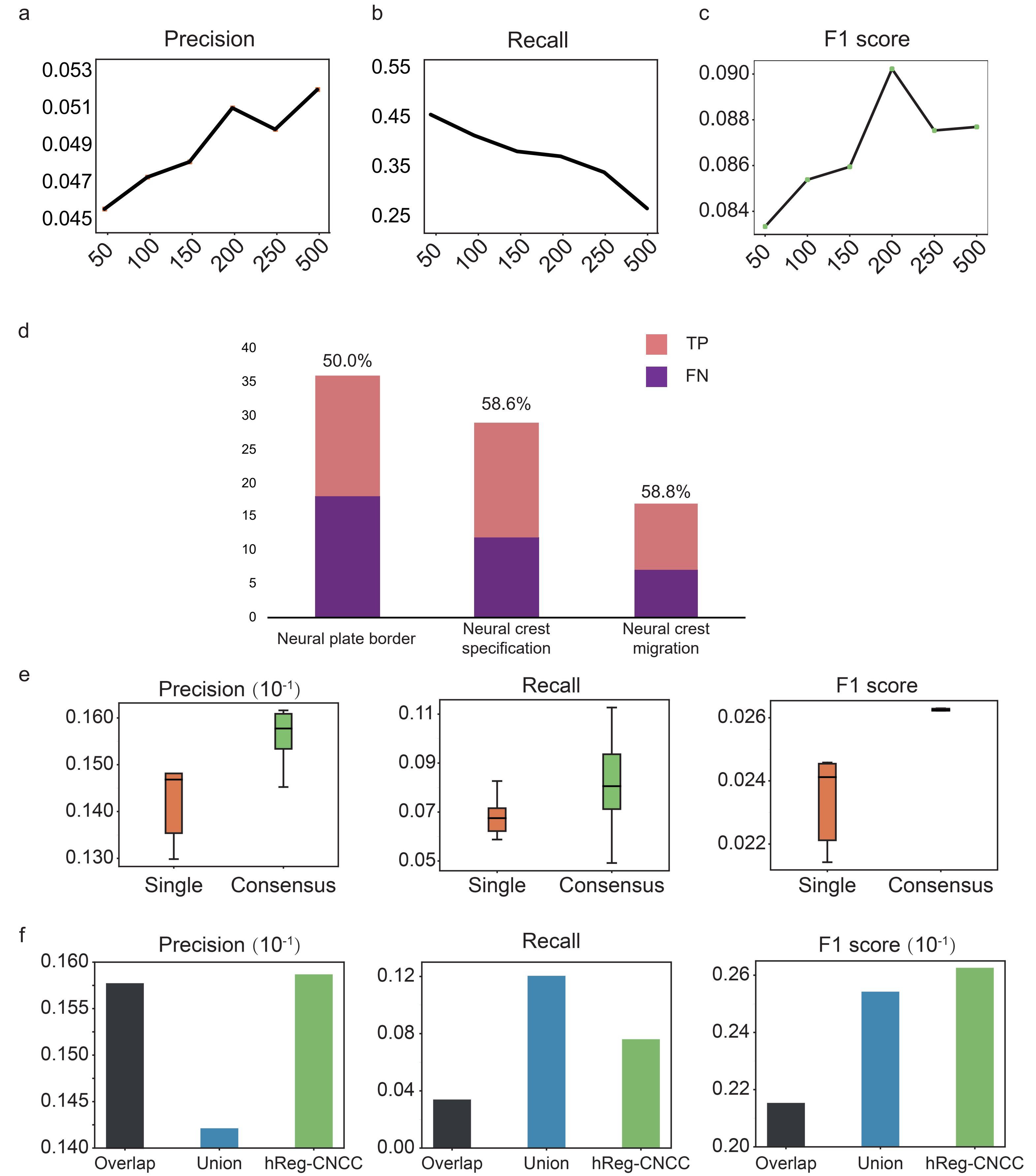

Supplementary Figure 1. Validation of hReg-CNCC shows consensus optimization obtain higher-quality regulatory network. (a-c). Precision, Recall, and F1 scores of consensus networks with different parameters (50, 100, 150, 200, 250, 500) (d). Coverage of three CNCC pathway functions in hReg-CNCC. (e). With chick -GRN as gold standard, consensus optimization achieves significantly higher precision, recall, and F1 measure than single networks. N=6 for consensus optimization and N=6 for single networks. (f). Consensus optimization outperforms the naive union and intersection methods in precision, recall, and F1 measure.

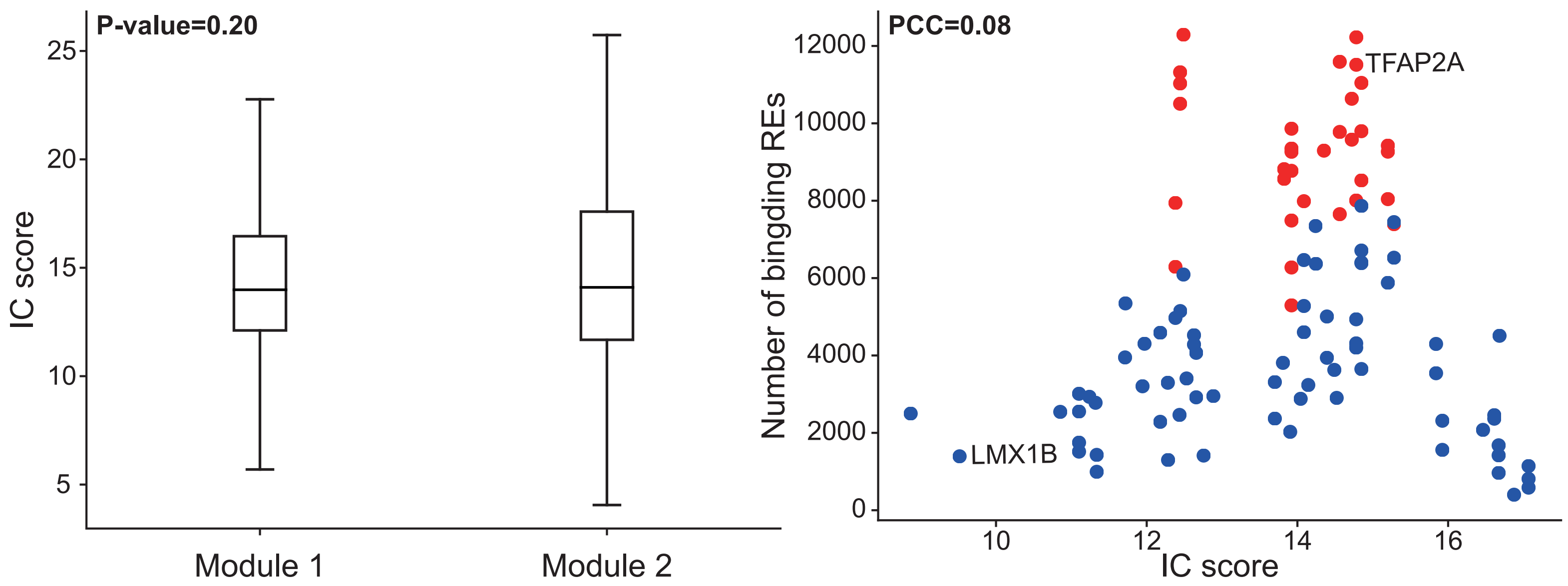

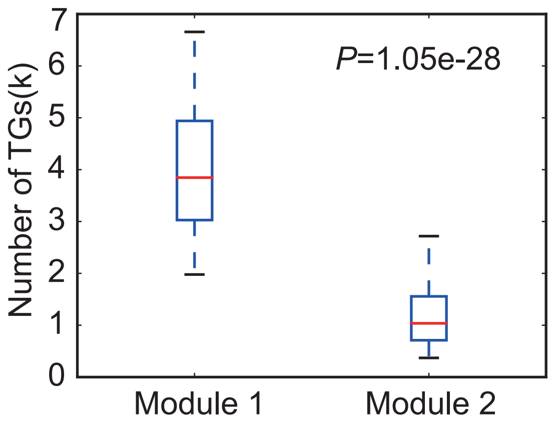

d

TFs	Tissues	TFs	Tissues
SIX1	skeletal muscle	DLX1	in vitro differentiated neuronal progenitor
SIX2	in vitro differentiated fibroblast	MSX1	Schwann cell
RFX3	prostate epithelial cell	BARX1	spleen
ZIC2	caudate	MSX2	embryoid body differentiation 2
ZIC1	caudate;cerebellar vermis	TCF7	thalamus subthalamic nucleus
HEY2	heart atrium	TCF7L2	thalamus
SP3	thalamus lateral nuclei	LEF1	thalamus subthalamic nucleus
ETS1	T cell	SIX4	corpus callosum
ETV1	cerebellar vermis	SOX4	hepatic stellate cell Ito cell
ETV5	adipose tissue subcutaneous	ELK3	msc to chondrocyte differentiation day 3
MYC	cultured breast epithelial cell	ETV3	myeloid dendritic cell
ARNT2	adrenal gland cortex(4)	MYBL2	trophoblast cell
FOXO3	prostate epithelial cell	AR	cerebral cortex
TWIST1	msc to chondrocyte differentiation day 7	EBF1	adipose tissue omental
TWIST2	chondrocyte	SMAD3	colon epithelial cell
EMX2	urethra	MAZ	prostate epithelial cell
		PATZ1	prostate epithelial cell

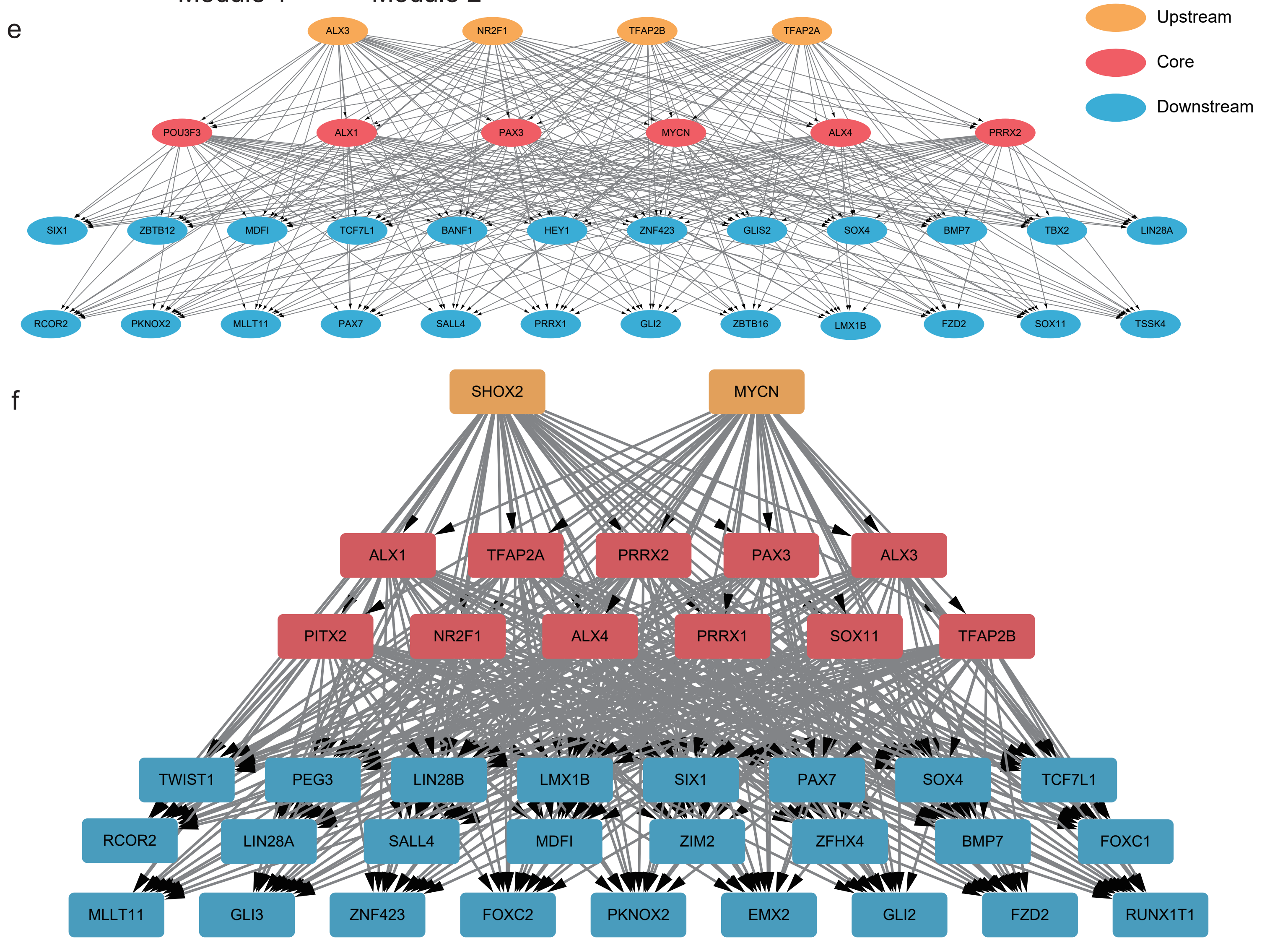

Supplementary Figure 2. Module classification is unrelated to motif information content and TFs in Module 1 show broader regulation while TFs in Module 2 show specific regulation, which is consistent with hReg-CNCC -H9. (a) Boxplot of information content (IC score) of TFs in Module 1 and Module 2. N=72 for Module 1 and N= 103 for Module 2. (b). Scatter plot shows no correlation between IC score and number of hReg-CNCC-predicted bound REs of 103 TF (32 Module 1 TFs and 71 Module 2 TFs). (c). The number of TGs regulated by TFs in Module 1 is significantly larger than that of TFs in Module 2. N=72 for Module 1 and N= 103 for Module 2. (d). TFs in Module 2 and associated tissues. (e). Dense TF network extracted from all TF set. (F). Dense TF network of hReg-CNCC-H9.·

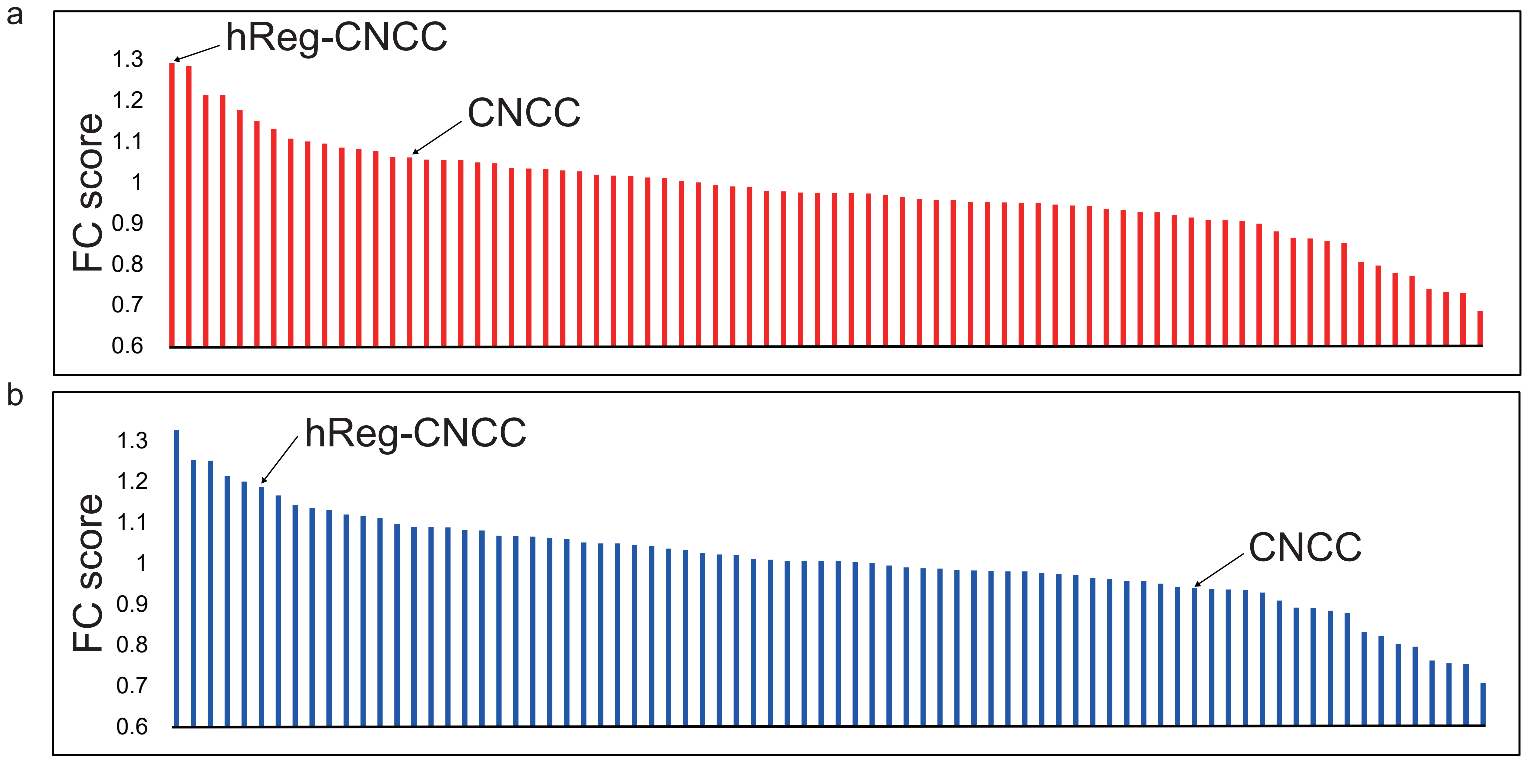

Supplementary Figure 3. The SNPs of facial morphology are more enriched in hReg-CNCC than randomly generated SNPs. (a). The ranked FC score of facial GWAS SNPs in hReg-CNCC RE set, CNCC peak set, and other 76 tissues' peak sets. (b). The ranked FC score of SNPsnap generated SNPs in hReg-CNCC RE set, CNCC peak set, and other 76 tissues' peak sets.

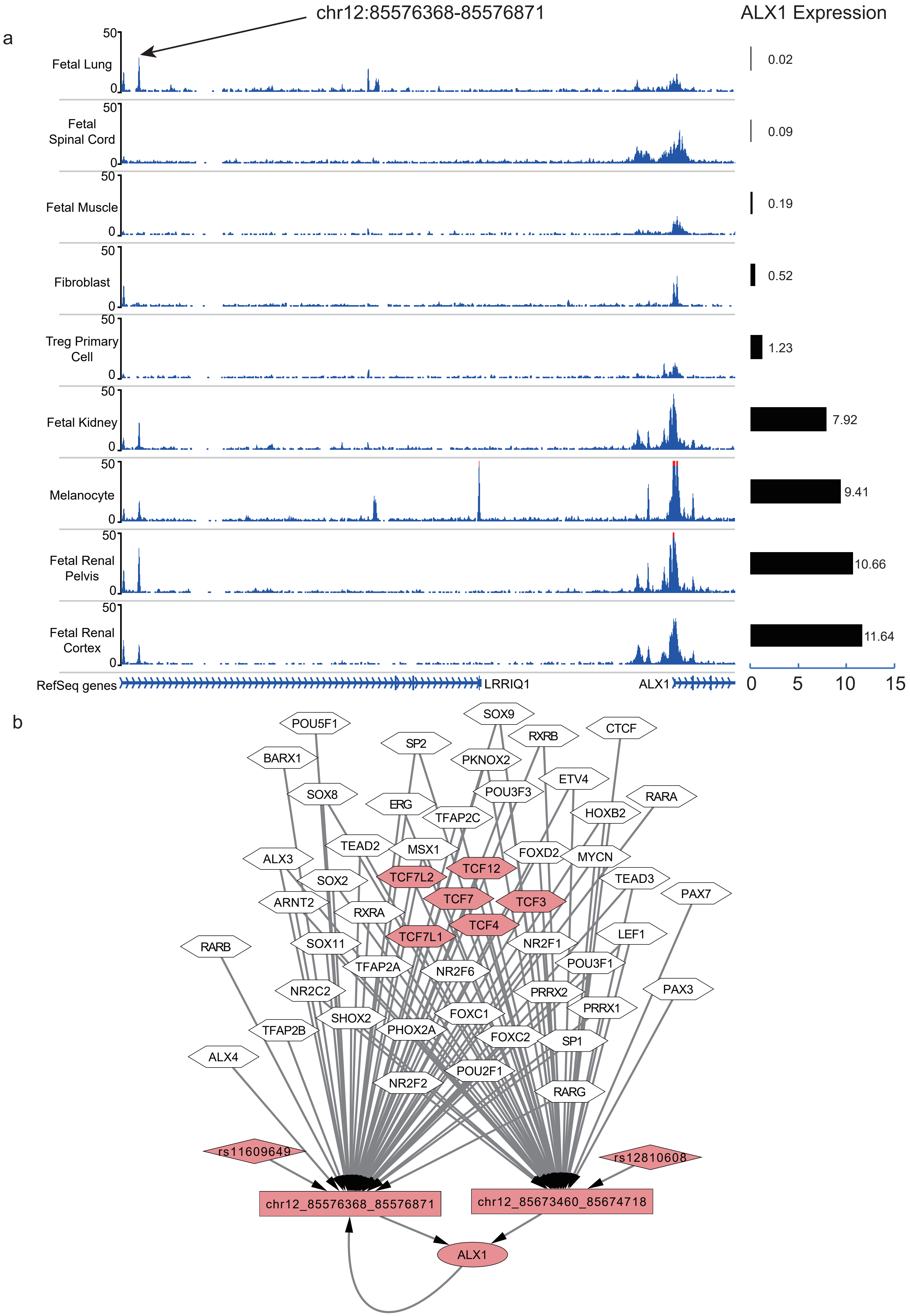

a
chr12:85576368-85576871
ALX1 Expression
Fetal Lung
0.02
Fetal Spinal Cord
0.09
Fetal Muscle
0.19
Fibroblast
0.52
Treg Primary Cell
1.23
Fetal Kidney
7.92
Melanocyte
9.41
Fetal Renal Pelvis
10.66
Fetal Renal Cortex
11.64
50
0
RefSeq genes
LRRIQ1
ALX1
0
5
10
15
b
POU5F1
SOX9
SP2
RXRB
CTCF
BARX1
PKNOX2
ETV4
SOX8
POU3F3
RARA
ERG
TFAP2C
HOXB2
TEAD2
MSX1
FOXD2
MYCN
ALX3
TCF7L2
TCF12
TEAD3
SOX2
TCF7
TCF3
PAX7
ARNT2
RXRA
TCF4
LEF1
RARB
SOX11
TCF7L1
NR2F1
TFAP2A
NR2F6
POU3F1
NR2C2
PRRX2
PAX3
SHOX2
FOXC1
PRRX1
TFAP2B
PHOX2A
FOXC2
SP1
ALX4
POU2F1
NR2F2
RARG
rs11609649
rs12810608
chr12_85576368_85576871
chr12_85673460_85674718
ALX1

Supplementary Figure 4. hReg-CNCC reveals correlated regulators of ALX1. (a). The accessibility of “chr12:85576368-85576871” is highly correlated with expression of ALX1 across diverse samples. (b). Sub-network related to ALX1 in hReg-CNCC. TCF cluster is important regulators to ALX1.

a

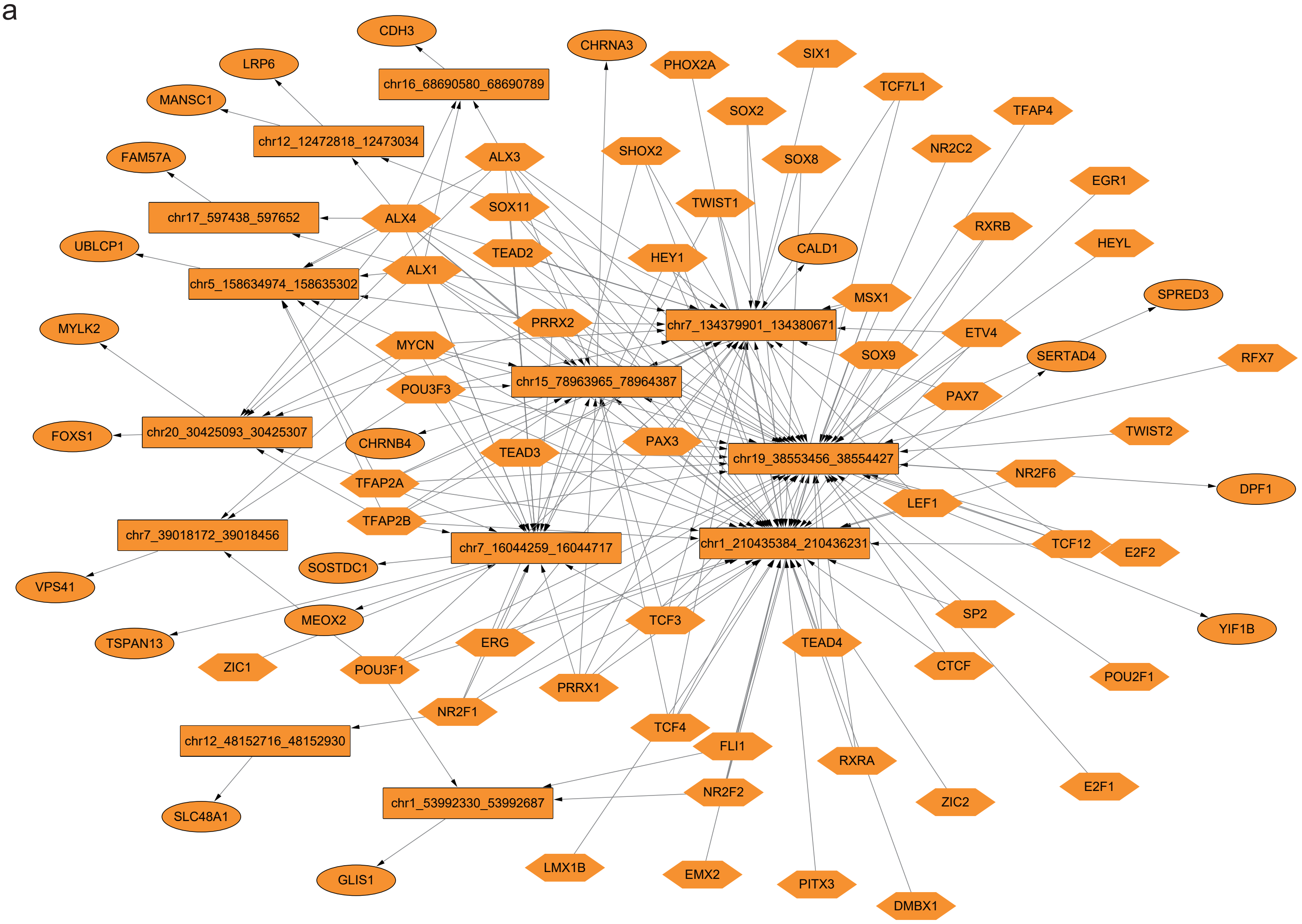

b

RE	SNP_Pvalue_Trait
chr1:210436017-210436248	rs12023563_0.02646_P106
chr12:48152927-48153096	rs12813049_0.1098_P112,rs1548518_0.01071_P117,rs76776452_0.002707_P6
chr15:78964052-78964383	rs12905641_0.0007578_P50
chr5:158635027-158635329	rs193261336_0.1025_P116,rs6556405_0.06327_P127
chr7:16044296-16045148	rs1029718_0.02248_P4,rs17169204_0.002407_P167

c

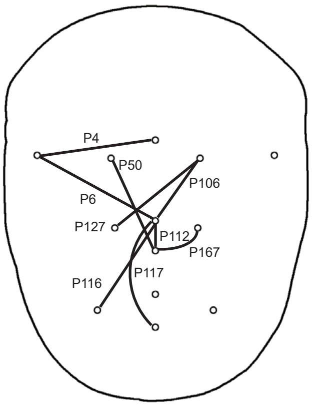

d

RE	Human Mean	Chimp Mean	Fold
chr20_30425093_30425307	6.473344666	0	647.3345
chr19_38553456_38554427	10.16852625	0.069969323	127.1553
chr7_134379901_13438067	15.81354307	0.137822416	106.9766
chr12_12472818_12473034	6.252109715	0.205441921	29.01993
chr15_78963965_78964387	5.184282538	0.174702023	28.06836
chr1_210435384_21043623	16.04605393	0.562641918	28.0211
chr5_158634974_15863530	11.75895331	0.567055574	20.37751
chr12_48152716_48152930	6.053316719	0.512560927	11.58394
chr7_16044259_16044717	8.06830975	0.831727427	9.585419
chr7_39018172_39018456	12.62150071	1.926436149	6.517902
chr16_68690580_68690789	3.400224537	0.730882755	4.589423
chr17_597438_597652	3.810254501	0.969611622	3.889556
chr1_53992330_53992687	5.292622778	1.564873507	3.360665

Supplementary Figure 5. Annotating Human accelerated regions (HARs) with hReg-CNCC reveals biological insights associated with face. (a). HARs were annotated by hReg-CNCC. (b). The HAR associated SNPs and face distances. (c). The HAR associated face distances are involved with nose area. (d). The average openness value in human and chimpanzee of 13 HARs in the annotated network.

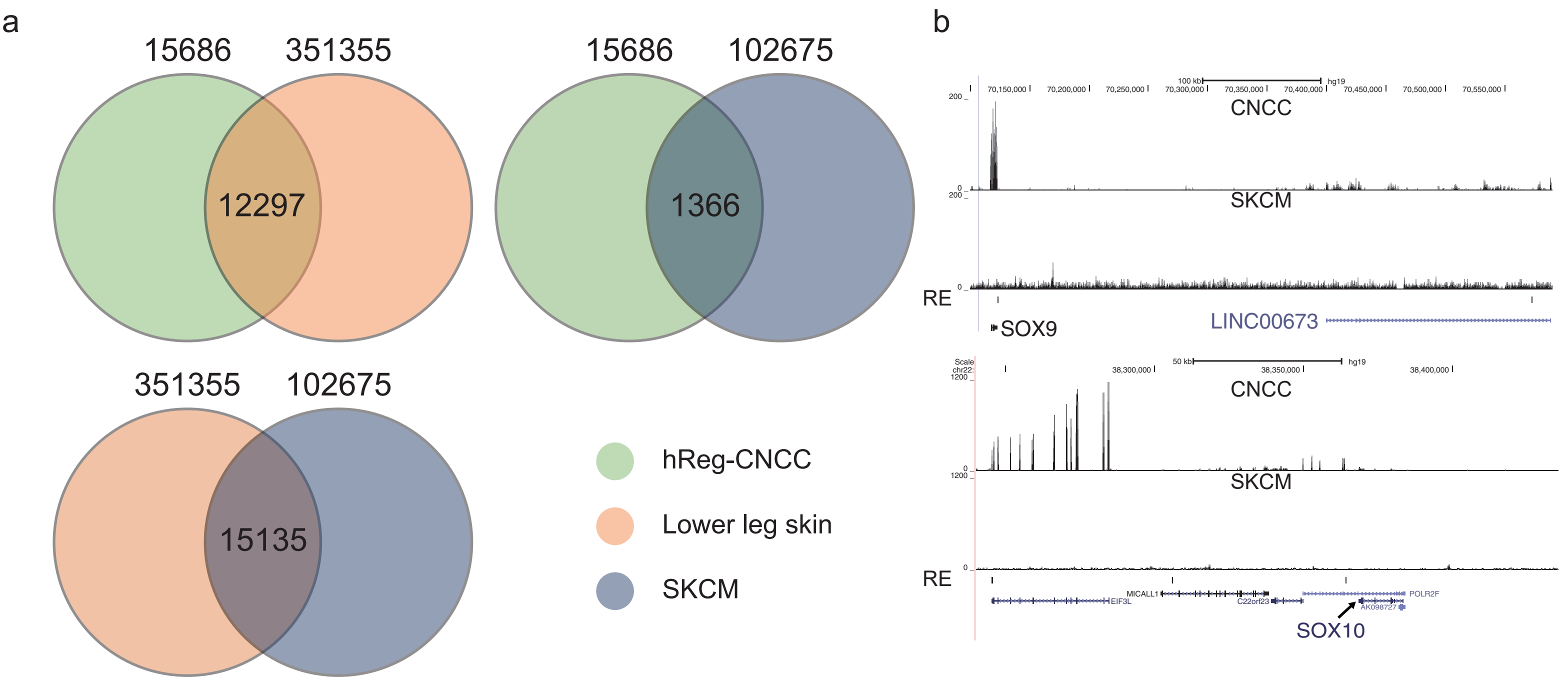

Supplementary Figure 6. Application of hReg-CNCC to study cancer (Skin Cutaneous Melanoma, SKCM) of CNCC derivatives. (a). Overlapping of REs in hReg-CNCC, ATAC-seq peaks of “lower leg skin”, and “SKCM”. (b). The REs regulating SOX9 and SOX10 were inactive in SKCM. We collected ATAC-seq peaks for SKCM of TCGA at https://gdc.cancer.gov/about-data/publications/ATACseq-AWG. To make comparison with normal tissue, we collected tissue of “lower leg skin” in ENCODE under accession ENCSR864IGD. We overlapped the 15,686 REs in hReg-CNCC with ATAC-seq peaks of “lower leg skin” and “SKCM” respectively. We found that 12,297 of the hReg-CNCC’s REs (78.39%) were also accessible in the normal “lower leg skin”, which indicated that the neural crest derivatives shared some of the epigenomic landscape of neural crest. However, only 1,366 REs (8.71%) in hReg-CNCC were accessible in “SKCM” (a). And the REs that regulated important NC TFs, such as SOX9/10, were all inactive in SKCM (b). This different overlapping ratios with hReg-CNCC between normal skin and skin cancer were consistent with the huge difference between normal skin and skin cancer (a). These observations showed that hReg-CNCC held the promise to study the pathology of cancer.
