# Supplementary material for: hReg-CNCC reconstructs a regulatory network in human cranial neural crest cells and annotates variants in a developmental context
Source: Commun Biol. 2021 Apr 6;4:442. doi: 10.1038/s42003-021-01970-0 (PMC8024315; doi:10.1038/s42003-021-01970-0)
Supplement: Supplementary file 3 — Description of Additional Supplementary Files [file 42003_2021_1970_MOESM3_ESM.pdf]

Description of Additional Supplementary Files

**File name:** Supplementary Data 1

**Description:** Paired RNA-seq and ATAC-seq data used for construction of hReg-CNCC.

**File name:** Supplementary Data 2

**Description:** The upstream TFs and REs regulating ALX1.

**File name:** Supplementary Data 3

**Description:** List of human paired gene expression and chromatin accessibility data used in regression of interaction strength.

**File name:** Supplementary Data 4

**Description:** 27 tissues used for control of fold change enrichment of face GWAS.

**File name:** Supplementary Data 5

**Description:** Source data underlying the graphs and charts presented in the main figures.
